# Forecasting the value of innovation in total knee arthroplasty care: A headroom approach

**DOI:** 10.1002/jeo2.70096

**Published:** 2024-12-18

**Authors:** Thomas M. Otten, Sabine E. Grimm, Bram Ramaekers, Alex Roth, Pieter Emans, Tim Boymans, Maarten Janssen, Ralph Jeuken, Manuela A. Joore

**Affiliations:** ^1^ Department of Clinical Epidemiology and Medical Technology Assessment (KEMTA) Maastricht University Medical Centre Maastricht The Netherlands; ^2^ Department of Orthopaedic Surgery CAPHRI School for Public Health and Primary Care, Maastricht University Medical Centre Maastricht The Netherlands

**Keywords:** cost saving, economic advantage, health technology assessment, knee arthroplasty, osteoarthritis

## Abstract

**Purpose:**

Total knee arthroplasty (TKA) is the standard treatment of end‐stage osteoarthritis. TKA is often used and, therefore, poses a healthcare and societal burden, which is likely to increase further. Headroom analyses evaluate a technology under development by making assumptions about its effectiveness. This article applies a headroom approach to forecast the potential value of innovations that improve TKA‐related care in the Netherlands in terms of cost‐effectiveness and surgeries avoided.

**Methods:**

A state‐transition model estimating lifetime direct health effects, healthcare‐ and societal costs and percentage of avoide d surgeries was developed. The model compared care as usual to five hypothetical interventions to calculate the headroom associated with (1) preventing the need for TKAs, (2) preventing the need for all TKA revisions, (3) postponing TKAs without quality‐of‐life loss, (4) preventing periprosthetic joint infections (PJIs) and (5) improving patient satisfaction.

**Results:**

Preventing the need for all TKAs amounted to €43,076 of headroom. Preventing the need for TKA revisions amounted to €2276 (5.8% of surgeries avoided), postponing TKAs by 5 years amounted to €7634 (32.4% of surgeries avoided), preventing PJIs amounted to €1187 (1.4% of surgeries avoided) and improving patient satisfaction amounted to €16,622 (0% of surgeries avoided). The headroom of each hypothetical intervention was highest in younger populations (<50 years of age).

**Conclusion:**

There is a headroom for improving TKA‐related care. Innovations to avoid or postpone TKA (i.e., joint‐preserving treatments) as well as those that improve patient satisfaction can be effective in maximizing the value for money and avoiding surgeries. Due to the decreasing average patient age, innovations to reduce revision rates and PJIs will become more valuable as these are most effective in younger patients. It is currently unclear how cost‐effectiveness considerations should be traded off against the prevention of surgery to reduce the increasing burden on the healthcare system.

**Level of Evidence:**

Level III economic evaluation/decision‐analytic model.

**Abbreviations**
AMP: amputation
ATD: arthrodesis
CAU: care as usual
LROI: Landelijke Registratie Orthopedische Interventies (Dutch Orthopaedic Registry)
OA: osteoarthritis
PJI: periprosthetic joint infection
QALY: quality‐adjusted life years
QoL: quality of life
TKA: total knee arthroplasty

## INTRODUCTION

Knee osteoarthritis (OA) is a degenerative joint disease with a lifetime prevalence of nearly 50% [39]. Because of its prevalence, knee OA was responsible for 0.5% of the overall healthcare costs in the Netherlands in 2019 [3]. When OA becomes severe, causing chronic pain and functional impairment, it may warrant a total knee arthroplasty (TKA) [28]. TKA is cost‐effective [16] and clinically effective [11], making it the established treatment for cases with end‐stage OA. In the Netherlands, the lifetime risk for TKA is 10%–23% for women and 6%–15% for men [1]. Accordingly, the Netherlands with a population of about 17.5 million in 2021 [43] registered 21,444 TKAs [18] in the same year. TKA places a high burden on healthcare systems in developed countries because of its widespread use. The volume of care in Germany, for example, is expected to further increase by 29% from 2023 to 2040 [48]. The greatest increase is anticipated among patients under the age of 70 [48].

The burden of TKA on individual patients is high: even without major complications, about one in five TKA patients is dissatisfied due to continued pain and limited function [17, 18]. Furthermore, rehabilitation from the surgery is demanding [32] and surgery bears the risk of periprosthetic joint infections (PJIs) [46]. Finally, the survival of TKAs is limited (87%–94% 10‐year survival) [18]. Revision TKAs resulting from failed TKAs are more expensive than primary TKAs and lead to worse health outcomes [6, 42]. The lifetime revision risk is highest in younger patients, the demographic in which the need for TKAs is expected to rise most [18]. The expected increase in the number of younger patients will, therefore, also mean an increase in the need for revision surgery [5, 48].

Care for patients eligible for TKA is continuously being improved through various interventions. Examples of these enhancements include weight loss programs [44], joint‐preserving treatments [27], osteotomy [37] and improved surgical techniques [4] during TKA. These interventions have wide‐ranging effects, such as postponing primary TKA [13], preventing revision TKA [18, 54] and reducing PJIs [26]. Collectively, these improvements contribute to both enhanced clinical outcomes and greater cost‐effectiveness in managing TKA.

A comparative assessment of the value of improving or preventing TKAs could be useful to prioritize research and development to improve TKA‐related care and counteract the rise in TKA volume. Headroom analysis as a form of early health technology assessment quantifies the value of an intervention under development by making assumptions about its effectiveness [9]. This specific form of analysis is aimed to inform research and development decisions based on the potential value of an intervention.

The aim of this article is to quantify the headroom of hypothetical technologies that offer various outcome improvements in late‐ and end‐stage OA care. These effects are reflected by five different hypothetical innovations that bring about five different improvements in outcomes in end‐stage OA treatment: (1) preventing the need for TKA and restoring quality of life (QoL) to that of a satisfied patient, (2) preventing revision TKA, (3) postponing TKAs without loss of QoL, (4) preventing PJIs and (5) improving patient satisfaction. The hypothesis is that all of the hypothetical innovations will have substantial value both in terms of health economic outcomes and capacity.

## METHODS

### Scope

The headroom analysis quantified the potential value for money and number and percentage of TKAs avoided for hypothetical interventions that provide different improvements in care for patients with severe OA who are about to undergo a TKA. Care as usual (CAU) was treated as the comparator and was defined as TKA surgery.

A state‐transition model in Microsoft Excel with a yearly cycle length was constructed. The analysis was conducted from the Dutch societal perspective. The model quantified direct healthcare and productivity costs and benefit. To capture all effects of the interventions, a lifetime horizon was applied. Costs were valued in Euros (€), adjusted for inflation to 2023 [14]. Health benefits were valued in quality‐adjusted life years (QALYs). As a measure of impact of the technologies on the healthcare system, the model quantified the number of surgeries (primary TKA, revision TKA, arthrodesis, amputations) that were avoided because of each intervention. The model applied an age distribution based on the Dutch Arthroplasty Register (Landelijke Registratie Orthopedische Interventies (Dutch Orthopaedic Registry) [LROI]) data [18]. The cost‐effectiveness threshold was 20,000€ per QALY. Costs and effects were discounted with a rate of 4.0% and 1.5%, respectively, in line with Dutch guidelines [24].

### Hypothetical interventions

There is scope for the development of new technologies that aim to improve TKA‐related care. To reflect these problems and provide an indication of where the societal value associated with a new technology is largest, the following hypothetical interventions are included:

❖ Intervention 1 is an intervention that replaces TKA flawlessly. Patients receiving intervention 1 receive the QoL of a satisfied TKA patient [18] for their entire life without any surgery. This intervention is meant to reflect the total headroom available for improvements around care for patients eligible for TKAs. All other interventions can be measured by how well they capture this maximum achievable headroom of intervention 1.
❖ Intervention 2 prevents the need for all revision TKA. In the model, patients receiving this intervention run no more risk of additional surgery. This intervention quantifies the maximum value of technology which aims at preventing or postponing TKA. Technological advancements, including enhanced surgical techniques and implants, have progressively evolved to minimize the necessity for revision TKA [18, 54].
❖ Intervention 3 postpones knee surgery by 5 years while with the same effect on QoL as standard TKA. This intervention is meant to capture the value of joint‐preserving treatments. Joint‐preserving procedures like lifestyle interventions, treatments for osteochondral defects and osteotomies can be used to postpone total knee arthroplasties in patients with early OA [13].
❖ Intervention 4 prevents PJIs and PJI‐related revisions. This intervention captures the headroom of preventing PJI‐related care because this care is more expensive than care related to aseptic revisions. Innovations to reduce PJIs vary widely and range from specific suture materials and surgical site antisepsis [26] to novel antimicrobial coatings of implants [41].
❖ Intervention 5 increases QoL for all patients to that of a satisfied patient. Since there is limited knowledge about what determines a patient's satisfaction with their TKA, this intervention determines how important a driver of societal value patient satisfaction is.

### Model structure

**Figure 1:**
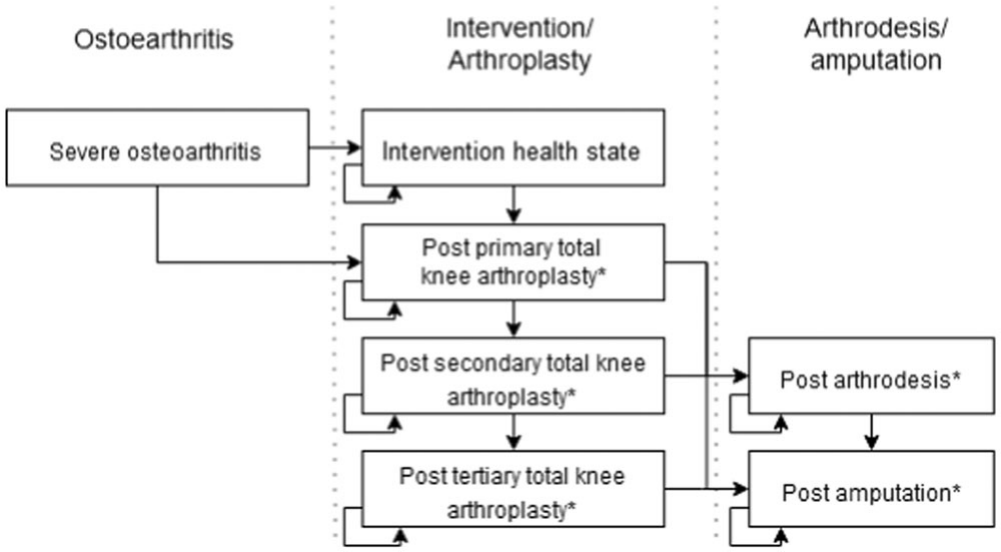
Model structure, all health states with * contain tunnel health states.

The model structure contains seven overarching health states (Figure 1): severe OA, an intervention health state (in which patients had their QoL restored due to an intervention), postprimary TKA, postsecondary TKA (revision TKA), posttertiary TKA (re‐revision TKA), postarthrodesis and postamputation. All patients moved to the postprimary TKA or the intervention health state in the first model cycle. Patients receiving CAU, intervention 2, 4 or 5 moved to the postprimary TKA health in the first model cycle. If patients received intervention 1, they moved to the intervention health state indefinitely. If patients received intervention 3, they moved from the intervention health state to the postprimary TKA health state at a constant rate so that the average patient received their TKA after 5 years. The model uses tunnel health states to account for changes in transition probabilities, QoL and costs over time. An illustration of the model structure with tunnel states can be found in Supporting Information S1: Appendix 1.

### Inputs

#### Transition probabilities

Patients in the model could experience seven different events: the hypothetical intervention, primary TKA, revision TKA, arthrodesis, amputation, PJI and death. All patients except patients receiving intervention 1 or intervention 3 received primary TKA in the first model cycle. TKA revisions were modelled to be age‐, time‐ and revision‐dependent based on LROI data: [18]

− Age‐dependence: Revision rates decrease with advancing age of primary TKA. For revisions from primary TKA, age‐dependent revision rates were implemented in 10‐year intervals [18].
− Time‐dependence: Revision rates decrease with time passed since receiving a TKA. The model included different exponential curves for early revisions (revisions in the first 3 years) and nonearly revisions (all years thereafter) to take changing revision rates over time into account [18].
− Revision dependence: Revision rates increase after the primary revision. The model included different revision rates for revisions from primary TKA and revision TKA (from secondary or tertiary TKA) [18].

Netherlands‐specific TKA revision rates which accounted for age, time and revision were not readily available. They, therefore, had to be extracted from graphs provided by the LROI report 2022 [20]. Kaplan–Meier curves for average revision rates, age‐specific revision rates and re‐revisions were extracted using the software ScanIt (v.2.0.8.0). Data were subsequently split up into the first 3 years and the years thereafter. Exponential survival curves were then constructed using R. These exponential survival curves were implemented in the economic model.

Arthrodesis and amputation were modelled to occur with a constant rate in all post‐TKA health states. Additionally, all revisions after the third TKA were assumed to be arthrodesis or amputations.

PJIs were modelled to occur after patients had received their primary TKA. PJIs were modelled to be time‐dependent and revision‐dependent [31]. General population mortality was applied based on data from the Central Bureau of Statistics of the Netherlands (CBS) [50].

Table 1 contains the transition probabilities applied in the model.

**Table 1:** Transition probabilities.

Category	Applied in (overarching health state)	Applied in (tunnel health state)	Yearly rate	Source
**Age** < **50**	**Postprimary TKA**	**1st, 2nd and 3rd year**	0.0242 (0.0220–0.0266)	LROI report 2022 [18]
		**4+ years**	0.0024 (0.0001–0.0083)	
**Age 50–59**	**Postprimary TKA**	**1st, 2nd and 3rd year**	0.0166 (0.0158–0.0175)	
		**4+ years**	0.0020 (0.0007–0.0037)	
**Age 60–69**	**Postprimary TKA**	**1st, 2nd and 3rd year**	0.0111 (0.0106–0.0116)	
		**4+ years**	0.0011 (0.0006–0.0019)	
**Age 70–79**	**Postprimary TKA**	**1st, 2nd and 3rd year**	0.0091 (0.0086–0.0096)	
		**4+ years**	0.0005 (0.0002–0.0011)	
**Age 80+**	**Postprimary TKA**	**1st, 2nd and 3rd year**	0.0064 (0.0059–0.0069)	
		**4+ years**	0.0001 (0.0000–0.0009)	
**Re‐revision**	**Postsecondary and posttertiary TKA**	**1st, 2nd and 3rd year**	0.0417 (0.0392–0.0443)	
		**4+ years**	0.0060 (0.0032–0.0099)	
**ATD**	**Postprimary, ‐secondary and ‐tertiary TKA**		0.0002 (0.0001–0.0003)	Gottfriedsen 2016 [22]
**AMP**	**Postprimary, ‐secondary and ‐tertiary TKA**		0.0002 (0.0001–0.0003)	Gottfriedsen 2016 [23]
**PJI**	**Postprimary TKA**	**1st and 2nd year**	0.0078 (0.0055–0.0105)	Kurtz et al. [31]
	**Postprimary TKA**	**3+ years**	0.0006 (0.0004–0.0008)	
	**Revision/ATD/AMP**	**1st and 2nd year**	0.0215 (0.0178–0.0255)	Blom [8], Cury [15] and Rodriquez [45]
	**Revision/ATD/AMP**	**3+ years**	0.0017 (0.0000–0.0228)	Kurtz et al. [31], Blom [8], Assumption

Abbreviations: AMP, amputation; ATD, arthrodesis; PJI, periprosthetic joint infection; TKA, total knee arthroplasty.

#### Health‐related QoL

The model used estimates from the LROI report 2022 [18] and published literature [19, 55, 57, 58] to calculate QoL. QoL was age‐adjusted according to values from Szende et al. [52]

Table 2 gives an overview of the most important QoL inputs.

**Table 2:** Quality of life inputs.

Health state	Tunnel state	Utility	Source
**Intervention 1 (pre‐TKA)**	Intervention health state	0.89	Based on high TKA patient satisfaction LROI report 2022 [18]
**Intervention 2 (pre‐TKA)**	Intervention health state	0.80	Based on average TKA patient satisfaction LROI report 2022 [18]
**Intervention 5 (postprimary TKA addition)**	Intervention health state	+0.06	Average increase in patient satisfaction if all ‘not satisfied’ patients have the QoL of ‘moderately satisfied’ patients [18]
**Postprimary TKA (Intervention 3)**	1st year	0.78 (0.78–0.78)	LROI report 2022 [18]
	2nd year	0.80 (0.80–0.80)	LROI report 2022 [18]
	3rd year	0.80 (0.80–0.80)	LROI report 2022 [18]
	4+ years	0.80 (0.80–0.80)	LROI report 2022 [18]
**Postprimary TKA**	1st year	0.78 (0.78–0.78)	LROI report 2022 [18]
	2nd year	0.80 (0.80–0.80)	LROI report 2022 [18]
	3rd year	0.80 (0.80–0.80)	LROI report 2022 [18]
	4+ years	0.80 (0.80–0.80)	LROI report 2022 [18]
**Postrevision TKA**	1st year	0.75 (0.71–0.79)	LROI report 2022 [18] and Woude, 2016 [57]
	2nd year	0.76 (0.72–0.80)	LROI report 2022 [18] and Woude, 2016 [57]
	3rd year	0.76 (0.72–0.80)	LROI report 2022 [18] and Woude, 2016 [57]
	3+ years	0.76 (0.72–0.80)	LROI report 2022 [18] and Woude, 2016 [57]
**Post‐ATD**	1st year	0.54 (0.24–0.84)	Wu, 2014 [58]
	2nd year	0.74 (0.52–0.66)	Wu, 2014 [58]
	2+ years	0.74 (0.52–0.66)	Wu, 2014 [58]
**Post‐AMP**	1st year	0.26 (0.08–0.24)	Ernstsson, 2021 [19], Wu, 2014 [58]
	2+ years	0.46 (0.44–0.48)	Ernstsson, 2021 [19]
**PJI**	n.a.	‐0.25 (0.11–0.43)	Walter, 2021 [55]
**Utility decrement prior to revision**	n.a.	‐0.10 (0.00–0.20)	LROI report 2022 [18], duration assumed 1 year

Abbreviations: AMP, amputation; ATD, arthrodesis; PJI, periprosthetic joint infection; TKA, total knee arthroplasty.

#### Healthcare costs

The model includes surgery costs and health state costs. Costs are sourced from the website opendisdata.nl [60], the iMTA costing tool [24] and the Dutch manual for costing studies in healthcare [29] as sources for inputs. Table 3 gives an overview of cost inputs. Health state costs were applied in each health state and included costs for follow‐up visits at different specialists as a result of having received treatment. In line with the notion of perfect effectiveness, no treatment cost for each of the five interventions was assumed.

**Table 3:** Cost inputs.

	Cost type	Cost	Source	Applied
Treatment cost	Primary TKA	€9650 (€8709–10,639)	Cost code 131999104 [60]	With every primary TKA
	Revision TKA	€14,520 (€13,104–16,007)	Cost code 192001008 [60]	With every revision TKA
	ATD	€12,810 (€11,561–14,122)	Cost code 192001019 [60]	With every arthrodesis
	AMP	€12,810 (€11,561–14,122)	Cost code 192001019 [60]	With every amputation
	Revision due to PJI	€46,320 (€41,803–51,065)	Cost code 192001008 [60], Bozic, 2005 [10]	With every PJI
Health state resource use	Pre‐TKA	€655 (€0–1310)	Combined, see Supporting Information S1: Appendix 1	With every TKA
	Postprimary or ‐revision TKA 1st year	€3071 (€0–641)	Combined, see Supporting Information S1: Appendix 1	Every year while in health state
	Postprimary or ‐revision TKA 2+ years	€119 (€0–239)	Combined, see Supporting Information S1: Appendix 1	Every year while in health state
	Post‐ATD 1st year	€3070 (€0–6141)	Combined, see Supporting Information S1: Appendix 1	Every year while in health state
	Post‐AMP 1st year	€5871 (€0–11,741)	Combined, see Supporting Information S1: Appendix 1	Every year while in health state
	Post‐ATD/AMP 2+ years	€120 (€0–239)	Combined, see Supporting Information S1: Appendix 1	Every year while in health state

Abbreviations: AMP, amputation; ATD, arthrodesis; PJI, periprosthetic joint infection; TKA, total knee arthroplasty.

#### Productivity costs

The friction cost method with a friction period of 85 days was implemented [33, 59]. Employment, wage and working hours were age‐adjusted according to data from the CBS [2, 53]. This productivity loss was implemented with every surgery.

### Analyses

#### Deterministic, probabilistic and uncertainty analyses

The results are calculated for each age category deterministically. The weighted average is calculated deterministically and probabilistically (1000 iterations). Deterministic sensitivity analyses and scenario analyses varying the effectiveness of the interventions to assess the influence of uncertainty on modelled results were conducted.

#### Headroom calculation

The headroom is the potential value of improving the current TKA practice with each of the five interventions. The formula for the headroom can be seen in Equation (1), where Effect_I_ and Cost_I_ Effect_C_ and Cost_C_ are the lifetime effects and costs of the intervention and the comparator, respectively. The cost‐effectiveness threshold is represented by λ. The model assumed perfect effectiveness for all interventions, meaning that the interventions would incur no additional costs and disutilities.

(1) $$\mathrm{Headroom}=\left({\mathrm{Effect}}_{I}-{\mathrm{Effect}}_{C}\right)*\lambda -({\mathrm{Cost}}_{I}-{\mathrm{Cost}}_{C}).$$

Equation: Headroom calculation

#### Avoided surgeries

The model quantifies the number of all surgeries (primary TKAs, revision TKAs, arthrodesis and amputations). To calculate the percentage of avoided surgeries, the number of surgeries in the intervention arm was divided by the number of surgeries in the comparator arm, which was then subtracted from one. For the number of avoided surgeries, the percentage of avoided surgeries was multiplied by the number of surgeries conducted in each age group in 2021 in the Netherlands [18]. The number of surgeries was discounted using the cost discounting rate.

#### Validation

The model was validated with the help of Consensus Health Economic Evaluation Reporting Standards, technical verification and external validation using multiple articles [16, 21, 47]. The results of the validation are reported in Supporting Information S2: Appendix 2. The article did not require IRB approval.

## RESULTS

### Headroom results

Table 4 contains the cost‐effectiveness results. The table shows the individual costs and health effects of each treatment in addition to the headroom of each intervention compared to CAU. The headroom of all five interventions was highest in the youngest age group. The headroom for Interventions 1, 2, 4 and 5 consistently decreased with advancing patient age. The headroom for intervention 2 also decreased from the <50 until the 71–80 age group but increased again in the oldest age group (>80).

**Table 4:** Base‐case cost‐effectiveness results (results by comparator and four interventions, every headroom in relation to the comparator, every result is deterministic, except for probabilistic weighted average results).

Age	Age distribution (%)	Comparator		1: Preventing all TKAs			2: Preventing all TKA revisions			3: Postponing TKA with 5 years			4: Preventing all PJIs			5: Improving patient satisfaction		
		QALY	Cost	QALY	Cost	Headroom	QALY	Cost	Headroom	QALY	Cost	Headroom	QALY	Cost	Headroom	QALY	Cost	Headroom
**<50**	**1**	21.13	€31,130	24.06	€0	€89,862	21.48	€23,609	€14,188	21.33	€22,408	€12,788	21.23	€28,228	€4914	22.45	€31,130	€26,404
**51–60**	**13**	16.78	€28,495	18.99	€0	€72,839	16.93	€24,343	€7018	16.89	€18,012	€12,670	16.82	€26,653	€2784	17.92	€28,495	€22,935
**61–70**	**34**	12.12	€17,154	13.70	€0	€48,715	12.19	€15,110	€3104	12.19	€11,251	€7162	12.14	€16,071	€1504	13.00	€17,154	€17,452
**71–80**	**40**	7.46	€13,812	8.46	€0	€33,804	7.49	€12,590	€1560	7.51	€8417	€6463	7.47	€13,053	€959	8.01	€13,812	€11,058
**>80**	**12**	3.84	€12,054	4.40	€0	€23,278	3.85	€11,330	€788	3.89	€5680	€7544	3.84	€11,525	€632	4.13	€12,054	€5839
**Weighted average results (deterministic)**		**9.96**	**€16,820**	**11.28**	**€0**	**€43,246**	**10.02**	**€14,934**	**€3026**	**10.02**	**€10,440**	**€7701**	**9.98**	**€15,816**	**€1382**	**10.67**	**€16,820**	**€14,303**
**Weighted average results (probabilistic)**		**9.92**	**€16,821**	**11.25**	**€0**	**€43,901**	**10.00**	**€14,941**	**€3271**	**10.01**	**€10,450**	**€7947**	**9.95**	**€15,834**	**€1432**	**10.64**	**€16,818**	**€14,280**

*Note*: Bold values denote the main results.

Abbreviations: PJI, periprosthetic joint infection; QALY, quality‐adjusted life years; TKA, total knee arthroplasty.

### Surgeries avoided

Table 5 shows the percentage of avoided TKAs and the number of avoided surgeries for each intervention [18]. Excluding Intervention 1, postponing all TKAs by 5 years would be most effective in patients aged 71–80 as the total number of avoided surgeries would be 2982. Postponing all TKAs by 5 years would be most efficient in patients aged older than 80 where 59.1% of TKAs would be avoided. The efficiency of intervention 3 increased with increasing patient age, while the efficiency of interventions 2 and 4 decreased with increasing patient age. The reason for this is that the main benefit of intervention 3 is to avoid primary TKA, while the main benefit of interventions 2 and 4 is to avoid revision surgeries. It was assumed that improving patient satisfaction would have no effect on the number of surgeries.

**Table 5:** Surgeries avoided.

Age	Patient distribution (%)	1: Preventing all TKAs		2: Preventing all TKA revisions		3: Postponing TKA by 5 years		4: Preventing all PJIs	
		% of TKAs avoided (%)	Number of TKAs avoided	% of TKAs avoided (%)	Number of TKAs avoided	% of TKAs avoided (%)	Number of TKAs avoided	% of TKAs avoided (%)	Number of TKAs avoided
**<50**	1	100.0	214	19.9	43	25.5	55	4.6	10
**51–60**	13	100.0	2788	12.4	345	24.9	693	2.9	82
**61–70**	34	100.0	7291	6.9	500	26.2	1907	1.6	119
**71–80**	40	100.0	8578	3.6	313	34.8	2982	0.9	73
**>80**	12	100.0	2573	1.7	43	59.1	1521	0.4	10
**Population‐level results (deterministic)**		**100.0**	**21,444**	**5.8**	**1244**	**32.4**	**6,47**	**1.4**	**293**
**Population‐level results (probabilistic)**		**100.0 (100.0–100.0)**	**21,444 (21,444–21,444)**	**6.0 (5.9–6.1)**	**1289 (1272–1303)**	**32.8%(32.8–32.9)**	**7036 (7025–7044)**	**1.4 (1.4–1.5)**	**304 (294–320)**

*Note*: Bold values denote the main results.

Abbreviations: PJI, periprosthetic joint infection; TKA, total knee arthroplasty.

Detailed results of the base‐case and sensitivity analyses are presented in Supporting Information S1: Appendix 1.

## DISCUSSION

The analyses result in differing conclusions when considering different outcomes. The maximum achievable headroom for preventing the need for all TKA surgeries was €43,901. This was best captured by improving patient satisfaction (€14,280), followed by postponing TKAs (€7947). In contrast, to avoid surgeries, next to preventing the need for all TKAs, postponing TKAs was by far the most effective intervention to avoid TKAs surgeries (32.4% of surgeries avoided), followed by reducing revisions (5.8% of surgeries avoided) and reducing PJIs (1.4% of surgeries avoided). It was assumed that there would be no benefit in avoiding surgeries from improving patient satisfaction.

The choice of where (target population) and how (intervention type) to innovate depends on whether value creation is desired in terms of value for money or reducing healthcare pressure. If maximizing value is the main aim, developing an intervention to postpone TKA or improve patient satisfaction would be preferable. The value for money of an innovation which prevents TKA revisions and PJIs is limited even if it would be perfectly effective as assumed here. However, targeting these interventions (preventing revision TKAs and PJIs) at subgroups that are at a higher risk of revision may be a way to ensure cost‐effectiveness. Specifically, younger patients and those suffering from obesity, diabetes and smokers are at risk of experiencing PJIs and TKA revisions, making them potentially cost‐effective targets for these interventions [12, 41, 46]. The headroom of all interventions is highest in the youngest patients as they benefit from the interventions the longest. The fact that the relative patient distribution is shifting toward younger patients in the coming years [18] means that the market size of the most valuable application of all innovations targeted will increase [34, 48]. All innovations explored here would, therefore, gain value in the future. At the same time, the health economic perspective of the model does not capture all benefits of the interventions.

If reducing pressure on the healthcare system is the main aim, developing an intervention to reduce TKA revisions especially for young populations or to postpone primary TKA would bring most benefit. Innovations with effects corresponding to the ones that were modelled could also serve other, broader and more difficult‐to‐capture societal aims: examples for this are that TKA care may improve independent living and thus reduce the pressure on long‐term care facilities. Furthermore, reducing the number of PJIs could be one of many necessary approaches to slow down the development of antibiotic‐resistant bacteria. Such broader societal aims are difficult to capture within an economic analysis.

There are no other articles that compare the cost‐effectiveness of different hypothetical innovations for TKA care. Three publications on headroom analyses concerning innovations at an earlier stage of knee surgery exist [36, 38, 56]. All three publications were about technologies implemented before TKA and did not model multiple iterations of age‐, revision‐ and time‐dependent knee arthroplasty revisions. Because this was the main mechanism of the article, their results are not comparable to the results of the model. Rovers [47], George [21] and Dakin [16] calculate the consequences of postponing TKA. All three articles agree that delaying TKA even with strong conservative assumptions (no costs in the OA health state, no further utility deterioration of OA when left untreated, short duration of delay) is not cost‐effective without improvement of the patient's QoL. Supporting Information S2: Appendix 2 provides an overview of attempts to externally validate the model by emulating the settings from Rovers [47], George [21] and Dakin [16]. While precise health economic results differ vastly, the conclusion that postponing TKA without QoL improvement is not cost‐effective would be the same.

The use of registry data can be seen as a limitation of this article. Confounding patient (i.e., OA severity, gender) and treatment characteristics (i.e., prosthesis/fixation type) [18] have not been taken into account and could bias the results. Exploration of these characteristics could further reveal groups and treatments in which innovation would be particularly valuable. A further limitation of this article is that the model also does not take into account that TKAs, revisions and PJIs all slightly increase mortality [25], long‐term care costs and long‐term employment postsurgery [51] especially for younger patients [35]. Because the modelled interventions reduce the incidence of TKAs and PJIs, the inclusion of additional costs and effects would only exacerbate the patterns that were demonstrated in these analyses. Including mortality would, therefore, be unlikely to change the conclusions. Similarly, while it is likely that patient satisfaction (i.e., Intervention 5) influences revision rates, this could not be considered because sufficient evidence to inform such analyses were unavailable. Finally, information on primary care resource use (i.e., physiotherapy, general practitioner) was largely unavailable. Similar limitations have also been found in other recent articles concerning knee surgery [7]. Inputs concerning health state resource use were, therefore, largely based on expert opinion and assumptions.

Additional research should be aimed at currently unavailable information. Considering that OA and TKA have a high prevalence and play a substantial role in the costs of a healthcare system [20], research into currently unavailable information (i.e., resource use, link of patient satisfaction to TKA revision rates) is relevant. From an innovator's point of view, this analysis could be augmented by adding a return‐on‐investment calculation to the current analysis. This would allow for predictions of possible profits given future changes in patient distribution. Finally, this analysis has been conducted from a Dutch perspective. However, with healthcare resources being limited, the incidence of OA is rising and the use of TKAs increasing similar patterns to what is observed in the Netherlands can be observed in many developed countries [30, 40, 49]. However, differences in reimbursement systems, including whether countries use value‐based healthcare at all or differences in cost‐effectiveness thresholds likely drastically change the headroom. Additional research could investigate the value of improving TKA‐related care in other countries.

## CONCLUSION

Innovations to avoid or postpone TKA (i.e., joint‐preserving treatments) as well as those that improve patient satisfaction have the highest headroom and may be effective at avoiding surgeries. Due to the decreasing average patient age, innovations to reduce revision rates and PJIs will become more valuable as these are most effective in younger patients.

## AUTHOR CONTRIBUTIONS

All authors contributed to the conception and planning of the article. Thomas M. Otten implemented the analysis and wrote the initial manuscript under the supervision of Bram Ramaekers, Sabine E. Grimm and Maarten Janssen. All authors consulted on the final manuscript.

## CONFLICTS OF INTEREST STATEMENT

A. R. is an employee and stockholder of Avalanche Medical BV. P. E. has acted as a paid consultant for DSM Biomedical, holds stock for Chondropeptix and Avalanche Medical and has supported research for DePuySynthes, Atro‐Meical and Hy2Care. The remaining authors declare no conflict of interest.

## ETHICS STATEMENT

The authors have nothing to report.

## Supporting information

Supporting information.

Supporting information.

## Data Availability

Data sharing is not applicable to this article as no new data were created or analyzed in this study.
