# Supplementary material for: Forecasting the value of innovation in total knee arthroplasty care: A headroom approach
Source: J Exp Orthop. 2024 Dec 18;11(4):e70096. doi: 10.1002/jeo2.70096 (PMC11653941; doi:10.1002/jeo2.70096)
Supplement: Supplementary file 1 — Supporting information. [file JEO2-11-e70096-s002.docx]

## Appendix 1 – Additional inputs

Health state resource use

Background resource costs (Table 1) was multiplied with background resource use (Table 2). Table 3 presents the health state costs per applied period.

| **Health state costs** | **Cost** | **Source** |
| --- | --- | --- |
| **GP visit** | €40 (€36 - €44) | Huisarts standard consult^19^ |
| **Orthopaedic specialist visit** | €140 (€126 -€154) | Combined: medisch specialist, algemeen ziekenhuis, academisch ziekenhuis consult^19^ |
| **Physical therapy** | €41 (€38 - €46) | Fysiotherapie consult^19^ |
| **Occupational therapy** | €41 (€38 - €46) | Occupational therapy consult^19^ |
| **Prosthesis (amputation)** | €2,800 (€2526 - €3087) | Kosten Bad/Zwemprothese ^20^ |
| **Rehabilitation professional** | €202 (€182 - €222) | Rehabilitation professional consult^19^ |

*Table 1 Health state resource costs*

|  | **GP visit** | | **Orthopedic specialist visit** | | **Physical therapy** | | **Occupational therapy** | | **Rehabilitation professional** | |
| --- | --- | --- | --- | --- | --- | --- | --- | --- | --- | --- |
|  | **Frequency** | **Source** | **Frequency** | **Source** | **Frequency** | **Source** | **Frequency** | **Source** | **Frequency** | **Source** |
| **First year after joint-preserving treatment** | 2 (0-4) | Expert opinion | 1 (0-2) | Expert opinion | 4 (0-8) | Expert opinion | 1 (0-2) | Expert opinion | 1 (0-2) | Expert opinion |
| **Year before TKA** | 3 (0 - 6) | Expert opinion | 2 (0 - 4) | Expert opinion | 8 (0 - 16) | Expert opinion | 1 (0 - 2) | Expert opinion | 0 (0 - 0) | Expert opinion |
| **First year after TKA, ATD, AMP (primary and revision)** | 2 (0 - 4) | Bulthuis^21^ | 1 (0 - 2) | Expert opinion | 12 (0 - 24) | Bulthuis^21^ | 1 (0 - 2) | Expert opinion | 2 (0 - 4) | Expert opinion |
| **More than 1 year after any intervention** | 0 (0 - 0) | Expert opinion | 0.2 (0 - 0.4) | Richtlijnendatabase^22^ | 2 (0 - 4) | Expert opinion | 0 (0 - 0) | Expert opinion | 0 (0 - 0) | Expert opinion |

*Table 2 Health state resource use*

|  | **Cost** |
| --- | --- |
| **First year after joint-preserving treatment** | € 792 (€ 0 - € 1,584) |
| **Pre TKA** | € 805 (€ 0 - € 1,610) |
| **First year after TKA, ATD, AMP (primary and revision)** | €1,193 (€ 0 – € 2,387) |
| **More than one year after any intervention** | €115 (€0 - €230) |

*Table 3 Health state costs*

Productivity costs

According to Dutch health economic guidelines^23^, we implemented the friction cost method with a friction period of 85 days^24^. Employment, wage and working hours were age-adjusted according to data from the CBS^25,26^. This productivity loss was implemented with every surgery.

All productivity costs per age group can be seen in table 5.

| **Age** | **Hours worked** | **Workforce participation** | **Productivity cost** | **Workdays missed** | **Productivity lost** |
| --- | --- | --- | --- | --- | --- |
| **Until 44** | 33.54 (27.29 - 40.43) | 0.88 (0.66 - 0.99) | € 28.82 (18.65 - 41.17) | 60.75 (48.60 - 72.90) | € 10,311.64 (8,084.33 - 12,538.96) |
| **45** | 33.39 (27.16 - 40.24) | 0.86 (0.66 - 0.98) | € 28.82 (18.65 - 41.17) | 60.75 (48.60 - 72.90) | € 10,094.78 (7,914.31 - 12,275.26) |
| **46-50** | 33.39 (27.16 - 40.24) | 0.86 (0.66 - 0.98) | € 29.77 (19.27 - 42.52) | 60.75 (48.60 - 72.90) | € 10,427.54 (8,175.19 - 12,679.89) |
| **51-54** | 33.39 (27.16 - 40.24) | 0.86 (0.66 - 0.98) | € 39.05 (25.27 - 55.78) | 60.75 (48.60 - 72.90) | € 13,678.05 (10,723.59 - 16,632.50) |
| **55** | 32.01 (26.04 - 38.58) | 0.74 (0.59 - 0.87) | € 39.05 (25.27 - 55.78) | 60.75 (48.60 - 72.90) | € 11,291.11 (8,852.23 - 13,730.00) |
| **56-60** | 32.01 (26.04 - 38.58) | 0.74 (0.59 - 0.87) | € 29.84 (19.31 - 42.62) | 60.75 (48.60 - 72.90) | € 8,628.09 (6,764.42 - 10,491.76) |
| **61-64** | 32.01 (26.04 - 38.58) | 0.74 (0.59 - 0.87) | € 29.32 (18.97 - 41.88) | 60.75 (48.60 - 72.90) | € 8,477.73 (6,646.54 - 10,308.92) |
| **65** | 20.85 (16.96 - 25.13) | 0.17 (0.14 - 0.21) | € 29.32 (18.97 - 41.88) | 60.75 (48.60 - 72.90) | € 1,978.33 (1,010.18 - 2,946.47) |
| **66-75** | 20.85 (16.96 - 25.13) | 0.17 (0.14 - 0.21) | € 27.18 (17.59 - 38.82) | 60.75 (48.60 - 72.90) | € 1,194.45 (936.45 - 1,452.45) |

*Table 4 Productivity costs*

Prosthetic Joint Infection cost

An article quantifying the cost of PJIs in the hip was used to calculate cost of PJIs after knee arthroplasty.^27^ The article resulted in a cost of PJIs in hip replacement that was 4.8 times that of primary hip replacements. The same factor was applied in this article resulting in a cost per PJI of €46,320. This cost was seen as realistic by an expert in PJIs who estimated the cost of PJI to be 4 to 5 times that of a primary TKA. The resulting uncertainty was further explored in sensitivity analyses.

Uncertainty

In cases where standard errors (SEs) were available for model inputs, these SEs were directly incorporated. When SEs were not available, a default variation of 20% was assumed. When the input was completely unavailable and had to be elicited through experts, a default SE of 100% was applied to reflect the increased uncertainty.
