# Supplementary material for: Forecasting the value of innovation in total knee arthroplasty care: A headroom approach
Source: J Exp Orthop. 2024 Dec 18;11(4):e70096. doi: 10.1002/jeo2.70096 (PMC11653941; doi:10.1002/jeo2.70096)
Supplement: Supplementary file 2 — Supporting information. [file JEO2-11-e70096-s001.docx]

**Appendix 2 – Quality of Reporting & Validation**

We conducted face validation by developing the model structure through regular through discussions between health economic researchers and orthopaedic surgeons. We used the CHEERS 2022 checklist^1^ to evaluate the quality of reporting of our article and the TECH-VER^2^ to internally validate the model. Further, we conducted several exercises for external validation: We compared the revision rates in the model with those of the LROI^3^ (dependent) and with the results of several articles^4–7^.

**CHEERS - 2022**^1^

|  | **Item** | **Guidance for Reporting** | **Reported in section** |
| --- | --- | --- | --- |
| **TITLE** | | |  |
| Title | 1 | Identify the study as an economic evaluation and specify the interventions being compared. | See ‘Title’ |
| **ABSTRACT** | | |  |
| Abstract | 2 | Provide a structured summary that highlights context, key methods, results and alternative analyses. | See ‘Abstract’ |
| **INTRODUCTION** | | |  |
| Background and objectives | 3 | Give the context for the study, the study question and its practical relevance for decision making in policy or practice. | See ‘Introduction’ |
| **METHODS** | | |  |
| Health economic  analysis plan | 4 | Indicate whether a health economic analysis plan was developed and  where available. | See Methods |
| Study population | 5 | Describe characteristics of the study population (such as age range, demographics, socioeconomic, or clinical characteristics). | See Methods |
| Setting and location | 6 | Provide relevant contextual information that may influence findings. | See Abstract, Introduction, and Methods, Dutch context |
| Comparators | 7 | Describe the interventions or strategies being compared and why chosen. | See Methods – Scope, Model structure |
| Perspective | 8 | State the perspective(s) adopted by the study and why chosen. | See Methods ‘Scope’ – Healthcare and productivity costs |
| Time horizon | 9 | State the time horizon for the study and why appropriate. | See Methods ‘Scope’ – Lifetime horizon |
| Discount rate | 10 | Report the discount rate(s) and reason chosen. | See Methods ‘Scope’ – 4.0% and 1.5% - In line with guidelines of the Zorginstituut Nederland |
| Selection of outcomes | 11 | Describe what outcomes were used as the measure(s) of benefit(s) and harm(s). | See Methods ‘Scope’ – Use of Health as outcome |
| Measurement of outcomes | 12 | Describe how outcomes used to capture benefit(s) and harm(s) were measured. | See Methods ‘Scope’ – QALYs |
| Valuation of outcomes | 13 | Describe the population and methods used to measure and value outcomes. | See Methods ‘Inputs’ – Health-related Quality of Life from literature |
| Measurement and valuation of resources  and costs | 14 | Describe how costs were valued. | See methods ‘Scope’ – costs valued in € |
| Currency, price date, and conversion | 15 | Report the dates of the estimated resource quantities and unit costs, plus the currency and year of conversion. | See Methods for Currency and Year of Conversion, See Appendix 1 for resource quantities and unit costs |
| Rationale and  description of model | 16 | If modelling is used, describe in detail and why used. Report if the model is publicly available and where it can be accessed. | See Methods ‘Scope’ – Use of state-transition model |
| Analytics and assumptions | 17 | Describe any methods for analyzing or statistically transforming data, any extrapolation methods, and approaches for validating any model used. | See Appendix 1 (HE Appendix) and 2 (Validation) |
| Characterizing heterogeneity | 18 | Describe any methods used for estimating how the results of the study vary for sub-groups. | See Methods, Inputs, Transition Probabilities – More information in HE Appendix |
| Characterizing  distributional effects | 19 | Describe how impacts are distributed across different individuals or adjustments made to reflect priority populations. | See methods - Analyses |
| Characterizing uncertainty | 20 | Describe methods to characterize any sources of uncertainty in the analysis. | See Methods – (probabilistic) Base-case analyses, sensitivity analyses |
| Approach to engagement with patients and others affected by the study | 21 | Describe any approaches to engage patients or service recipients, the general public, communities, or stakeholders (e.g., clinicians or payers) in the design of the study. | See Appendix 1 – Orthopaedic Surgeons were among the co-authors |
| **RESULTS** | | |  |
| Study parameters | 22 | Report all analytic inputs (e.g., values, ranges, references) including uncertainty or distributional assumptions. | See Appendix 1 – Inputs |
| Summary of main results | 23 | Report the mean values for the main categories of costs and outcomes of interest and summarise them in the most appropriate overall measure. | See Results – Table 2 |
| Effect of uncertainty | 24 | Describe how uncertainty about analytic judgments, inputs, or projections  affect findings. Report the effect of choice of discount rate and time horizon, if applicable. | See Results – Sensitivity Analysis, See Appendix 1 – Sensitivity Analysis & Figures |
| Effect of engagement with patients and others affected by the study | 25 | Report on any difference patient/service recipient, general public, community, or stakeholder involvement made to the approach or findings of the study | Not applicable |
| **DISCUSSION** | | |  |
| Study findings, limitations, generalizability, and current knowledge | 26 | Report key findings, limitations, ethical or equity considerations not captured, and how these could impact patients, policy, or practice. | See Discussion – Equity considerations not reported |
| **OTHER RELEVANT INFORMATION** | | | |
| Source of funding | 27 | Describe how the study was funded and any role of the funder in the identification, design, conduct, and reporting of the analysis |  |
| Conflicts of interest | 28 | Report authors conflicts of interest according to journal or  International Committee of Medical Journal Editors requirements. |  |

Husereau D, Drummond M, Augustovski F, de Bekker-Grob E, Briggs AH, Carswell C, Caulley L, Chaiyakunapruk N, Greenberg D, Loder E, Mauskopf J, Mullins CD, Petrou S, Pwu RF, Staniszewska S; CHEERS 2022 ISPOR Good Research Practices Task Force. Consolidated Health Economic Evaluation Reporting Standards 2022 (CHEERS 2022) Statement: Updated Reporting Guidance for Health Economic Evaluations. BMJ. 2022;376:e067975.

The checklist is Open Access distributed in accordance with the terms of the Creative Commons Attribution (CC BY 4.0) license, which permits others to distribute, remix, adapt and build upon this work, for commercial use, provided the original work is properly cited. See: [http://creativecommons.org/licenses/by/4.0/.](http://creativecommons.org/licenses/by/4.0/)

**TECH-VER**^2^

| Question | Expected answer | Answer | Documentation |
| --- | --- | --- | --- |
| Does the technology (drug/device, etc.) acquisition cost increase with higher prices? | Yes | Yes | Added cost of €10 per patient to intervention cost in the first cell of the surgery cost column in the sheet Cohort analysis – Intervention sheet. The intervention cost increased to €13,461. |
| Does the drug acquisition cost increase for higher weight or body surface area? | Yes | Not applicable because body weight and BSA are not included in the model. |  |
| Does the probability of an event, derived from an OR/RR/HR and baseline probability, increase with higher OR/RR/HR? | Yes | Yes, when probabilities are set higher, the event probability increases. | Set Parameters F63 to 1. Comparator TKAs increase to 1,427 TKAs, while Intervention TKAs remain the same. |
| In a partitioned survival model, does the progression-free survival curve or the time on treatment curve cross the overall survival curve? | No | Not applicable, the model is not a partitioned survival model but a state transition model. |  |
| If survival parametric distributions are used in the extrapolations or time-to-event calculations, can the formulae used for the Weibull (generalized gamma) distribution generate the values obtained from the exponential (Weibull or Gamma) distribution(s) after replacing/transforming some of the parameters? | Yes | Not applicable, only Exponential curves are implemented |  |
| Is the HR calculated from Cox proportional hazards model applied on top of the parametric distribution extrapolation found from the survival regression? | No | Not applicable, no hazard rates are applied in the model. |  |
| For the treatment effect inputs, if the model uses outputs from WINBUGS, are the OR, HR, and RR values all within plausible ranges? (Should all be non-negative and the average of these WINBUGS outputs should give the mean treatment effect) | Yes | Not applicable, WINBUGS was not used for the analysis of model inputs. |  |
| Calculate the sum of the number of patients at each health state | Should add up to cohort size | Adds up to cohort size | Cohort analysis sheets columns AC are implemented to check this. |
| Check if all probabilities and number of patients in a state are greater than or equal to 0 | Yes | Yes | Checked manually – Parameters sheet & Cohort analysis sheet |
| Check if all probabilities are smaller than or equal to 1 | Yes | Yes | Checked manually – Parameters sheet |
| Compare the number of dead (or any absorbing state) patients in a  period with the number of dead (or any absorbing state) patients in  the previous periods? | Should be larger | Yes | Checked manually – Cohort analysis sheet |
| In case of lifetime horizon, check if all patients are dead at the end  of the time horizon | Yes | Yes | Graphs summarized sheet – Column Q |
| Set all utilities to 1 | QALYs should equal LYs | QALYs equal LYs. | Set all utilities equal to 1 and removed all disutilities |
| Set all utilities to 0 | QALYs should equal 0 | QALYs equal 0 | Set all utilities to 0 and removed all utilities. |
| Decrease all state utilities simultaneously | Lower utilities should be accumulated at all times. | Lower utilities are accumulated at all times | Reduced health state utilitites by 10% QALYS are reduced to 9.288 and 9.332 in intervention and comparator arm respectively. The effect can also be seen in the cohort analysis sheets. |
| Set all costs to 0 | No costs will be accumulated in the model | Modelled costs are equal to 0 | Set all costs in parameters sheets to 0. Results sheet shows costs of 0 for intervention and comparator. |
| Put mortality rate to 0 | Patients never die | No patients die | Set mortality to 0 in cohort analysis sheet – Cohort analysis sheet shows that no patient dies. |
| Put mortality rate at extremely high | Patients die in the first few cycles | Patients die in the first few cycles | Set mortality rate at 0.99 in cohort analysis sheet – All patients are dead after 2 cycles. |
| Set the effectiveness-, utility-, and safety-related model inputs for all  treatment options equal | Same life-years and QALYs should be accumulated for all treatment at  any time | 0 incremental costs and QALYs | Set Assumption delay to 1, TKAdelay, QoLpreTKA, YrspreTKA to 0 |
| Change around the effectiveness-, utility- and safety-related model inputs between two treatment options | Outcomes should be reversed as well | Yes | Switched effectiveness inputs around. Utility and cost inputs were dependent on effectiveness inputs. |
| Check if the number of alive patients estimated at any cycle is in line with general population life-table statistics | At any given age, the percentage alive should be lower or equal in com- parison with the general population estimate | Yes | The only reason for mortality in the model is based on population mortality. |
| Check if the QALY estimate at any cycle is in line with general population utility estimates | At any given age, the utility assigned in the model should be lower or equal in comparison with the general population utility estimate | Yes | Comparison to Szende Dutch utility values |
| Set the inflation rate for the previous year higher | The costs (which are based on a reference from previous years) assigned at  each time will be higher | Yes | Must be checked manually as inflation rates are not directly implemented. |
| Calculate the sum of all ingoing and outgoing transition probabilities of a state in a given cycle | Difference of ingoing and outgoing probabilities at a cycle in a state times the cohort size will yield the change in the number of patients at that state in that cycle | Correct | Choose TKA1Y2 in the 7^th^ model cycle. 100% probability of leaving model cycle, 99.1% of patients moving there from TKA1Y1 model cycle (excl mortality) – numbers add up |
| Calculate the number of patients entering and leaving a tunnel state throughout the time horizon | Numbers entering = numbers leaving | Correct | Choose AMP_Y1 – all patients leave the health state every cycle. Therefore, the number of patients entering the health state is equal to the number of patients leaving the health state. |
| Check if the time conversions for probabilities were conducted cor- rectly. | Yes | Yes | See parameter sheet D235 to D341 |
| Increase the treatment acquisition cost | Costs accumulated at a given time will increase during the period when the treatment is administered | Correct | Added surgery cost of €10 for all patients in first cycle in the cohort analysis sheet. The cost increases to €13,554 |
| Set the mortality and incidence rates to 0 | Prevalence should be constant in time | All patients remain in the SevOA state indefinitely | There are no disease incidence rates |
| Check the incremental life-years and QALYs gained results. Are they in line with the comparative clinical effectiveness evidence of the treatments involved? | If a treatment is more effective, it generally results in positive incremental LYs and QALYs in comparison with the less-effective treatments | Yes | See validation sheet for comparison with LROI data |
| Check the incremental cost results. Are they in line with the treatment costs? | If a treatment is more expensive, and if it does not have much effect on other costs, it generally results in positive incremental costs | Broadly in line. Many patients receive a TKA, the incremental costs reflect the number of TKAs avoided in the intervention arm. Both arms have higher costs than the cost of single TKAs, which is as expected |  |
| Total life years greater than the total QALYs | Yes | Yes | See Results sheet (also page 1) |
| Undiscounted results greater than the discounted results | Yes | Yes | Remove discount rates – Costs and QALYs increase in favour of the comparator |
| Divide undiscounted total QALYs by undiscounted life years | Yes | Yes | Intervention: 0.76 🡪 close to 0.8 which is the highest utility that patients could possibly attain in the model |
| Subgroup analysis results: How do the outcomes change if the char- acteristics of the baseline change? | Better outcomes for better baseline health conditions, and worse outcomes for worse health conditions, are expected | Yes | Yes younger patients live longer and accumulate more QALYs. 55 year old patients accumulate 17 QALYs in comparison to about 10 for 69 year old patients (see above) |
| Could you generate all the results in the report from the model (including the uncertainty analysis results)? | Yes | Yes |  |
| Do the total life-years, QALYs, and costs decrease if a shorter time horizon is selected? | Yes | Yes | Results show lower incremental QALYs, costs and LYs when time horizon is decreased in parameters sheet. |
| Is the reporting and contextualization of the incremental results correct? | Yes | Yes |  |
| Are the reported ICERs in the fully incremental analysis non- decreasing? | Yes | Not applicable | We do not report a fully incremental analysis |
| If disentangled results are presented, do they sum up to the total results (e.g. different cost types sum up to the total costs esti- mate)? | Yes | Yes | See results sheet row 25 |
| Check if half-cycle correction is implemented correctly (total life- years with half-cycle correction should be lower than without) | The half-cycle correction implementation should be error-free. Also check if it should be applied for all costs, for instance if a treatment is adminis- tered at the start of a cycle, half-cycle correction might be unnecessary | Yes |  |
| Check the discounted value of costs/QALYs after 2 years | Discounted value = undiscounted/(1 + r)2 | Yes | Checked both in cohort analysis sheet |
| Set discount rates to 0 | The discounted and undiscounted results should be the same | Yes | Checked in cohort analysis sheet |
| Set mortality rate to 0 | The undiscounted total life-years per patient should be equal to the length of the time horizon | Yes | Set mortality to 0 in cohort analysis sheet, checked in results sheet |
| Put the consequence of adverse event/discontinuation to 0 (0 costs and 0 mortality/utility decrements) | The results would be the same as the results when the AE rate is set to 0 | Yes | Cohort analysis sheet Column AK calculation – incidence is multiplied by disutility. Setting either of them to 0 will remove the consequences of PJI |
| Divide total undiscounted treatment acquisition costs by the average duration on treatment | This should be similar to treatment-related unit acquisition costs | Yes | Treatment lasts one cycle |
| Set discount rates to a higher value | Total discounted results should decrease | Yes | Results indeed |
| Set discount rates of costs/effects to an extremely high value | Total discounted results should be more or less the same as the discounted  results accrued in the first cycles | Yes | Set discount rates to 500% results resemble results when only taking first model cycle into account |
| Put adverse event/discontinuation rates to 0 and then to an extremely high level | Less costs and higher QALYS/LYs when adverse event rates are 0, higher costs and lower QALYS/LYs when AE rates are extreme | Yes | Setting PJI incidence to 0 🡪 0 PJI disutility. PJI disutility of 1.2 to 2 for PJI incidence of 0.5 |
| Double the difference in efficacy and safety between the new inter- vention and comparator, and report the incremental results | Approximately twice the incremental effect results of the base case. If this is not the case, report and explain the underlying reason/mechanism | No | Intervention 1: Not possible to avoid more than 100% of TKAs.  Intervention 2: The benefits of postponing TKA are not linear. (revision rates don’t decrease linearly but only every 10 years, mortality doesn’t increase linearly).  Intervention 3 & 4: Not possible to double as revision rates are already reduced by 100%. |
| Do the same for a scenario in which the difference in efficacy and safety is halved | Approximately halve of the incremental effect results of the base case. If this is not the case, report and explain the underlying reason/mechanism | Partially | Intervention 1: True  Intervention 2: The benefits of postponing TKA are not linear. (revision rates don’t decrease linearly but only every 10 years, mortality doesn’t increase linearly).  Intervention 3 & 4: Approximately true, however non-linear revision rates slightly alter the results from what would be expected |
| Are all necessary parameters subject to uncertainty included in the OWSA? | Yes | Yes |  |
| Check if the OWSA includes any parameters associated with joint uncertainty (e.g. parts of a utility regression equation, survival curves with multiple parameters) | No | No, there are no utility regression equations or survival curves with multiple parameters |  |
| Are the upper and lower bounds used in the one-way sensitivity analysis using confidence intervals based on the statistical distri- bution assumed for that parameter? | Yes | Not always, sometimes bounds were unavailable. In that case 20% of the original parameter was used. |  |
| Are the resulting ICER, incremental costs/QALYs with upper and lower bound of a parameter plausible and in line with a priori expectations? | Yes | No, there are no real expectations for this calculations as it is not explored so far. OWSA results are not unexpected |  |
| Check that all parameters used in the sensitivity analysis have appropriate associated distributions – upper and lower bounds should surround the deterministic value (i.e. upper bound ≥ mean ≥ lower bound) | Yes | Yes |  |
| Standard error and not standard deviation used in sampling | Yes | Yes |  |
| Lognormal/gamma distribution for HRs and costs/resource use | Yes | Yes | See parameters sheet |
| Beta for utilities and proportions/probabilities | Yes | Yes | See parameters sheet |
| Dirichlet for multinomial | Yes | Not applicable |  |
| Multivariate normal for correlated inputs (e.g. survival curve or regression parameters) | Yes | Not applicable |  |
| Normal for other variables as long as samples do not violate the requirement to remain positive when appropriate | Yes | Not applicable |  |
| Check PSA output mean costs, QALYs, and ICER compared with the deterministic results. Is there a large discrepancy | No | No large discrepancy, very similar results |  |
| If you take new PSA runs from the Microsoft Excel model do you  get similar results? | Yes | Yes |  |
| Check the correlation between two PSA results (i.e. costs/QALYs  under the SoC and costs/QALYs under the comparator) | Should be very low | Checked for multiple results, all correlations close to 0 or 0. |  |
| If a certain seed is used for random number generation (or previously  generated random numbers are used), check if they are scattered  evenly between 0 and 1 when they are plotted | Yes | Not applicable |  |
| Compare the mean of the parameter samples generated by the model  against the point estimate for that parameter, use graphical methods  to examine distributions, functions | The sample means and the point estimates will overlap, the graphs will be  similar to the corresponding distribution functions (e.g. normal, gamma,  etc.) | Yes |  |
| Check if sensitivity analyses include any parameters associated with  methodological/structural uncertainty (e.g. annual discount rates,  time horizon) | No | No (except for discount rates for DSA) | Check parameter sheet |
| Value of information analysis if applicable: Was this implemented  correctly? | Yes | N/A |  |
| Which types of analysis? Were aggregated parameters used? Which  parameters are grouped together? Does it match the write-up’s  suggestions? | Yes | N/A |  |
| Is EVPI larger than all individual EVPPIs? | Yes | N/A |  |
| Is EVPPI for a (group of) parameters larger than the EVSI of that  (group) of parameter(s)? | Yes | N/A |  |
| Are the results from EVPPI in line with OWSA or other parameter  importance analysis (e.g. ANCOVA)? | Yes | N/A |  |
| Did the electronic model pass the black-box tests of the previous  verification stages in all PSA iterations and in all scenario analysis  settings? | Yes |  |  |
| Check if all sampled input parameters in the PSA are correctly  linked to the corresponding event/state calculations | Yes |  |  |

**External validation**

The comparison of the revision rate as applied by our model compared to the LROI data is provided in the table below.

| **Duration** | **Average** | | **Re-revision** | | **Revision age <50** | | **Revision age 50-59** | | **Revision age 60-69** | | **Revision age 70-79** | | **Revision age >80** | |
| --- | --- | --- | --- | --- | --- | --- | --- | --- | --- | --- | --- | --- | --- | --- |
|  | **Observed** | **Calculated** | **Observed** | **Calculated** | **Observed** | **Calculated** | **Observed** | **Calculated** | **Observed** | **Calculated** | **Observed** | **Calculated** | **Observed** | **Calculated** |
| **1** | 1.0% | 1.11% | 5.6% | 4.2% |  | |  | |  | |  | |  | |
| **3** | 3.3% | 3.30% | 12.0% | 12.0% |  |  |  |  |  |  |  |  |  |  |
| **5** | 4.2% | 4.08% | 15.3% | 15.1% |  |  |  |  |  |  |  |  |  |  |
| **8** |  |  | 18.2% | 19.6% |  |  |  |  |  |  |  |  |  |  |
| **10** | 5.5% | 5.99% |  | |  |  |  |  |  |  |  |  |  |  |
| **13** | 6.0% | 7.12% |  |  | 15.8% | 15.8% | 10.3% | 10.3% | 6.6% | 6.5% | 4.2% | 4.2% | 2.4% | 2.3% |

Table 7 Comparison of observed LROI data and calculated data

To compare our results to similar published literature we attempted to imitate analyses from other authors: Gademan calculated the revision TKAs avoided by delaying TKA by 5 years over a 10-year time horizon. Revision rates were stratified by age, sex and fixation type. Their analyses were also based on LROI data. To imitate Gademan’s analyses, we set our time horizon to 10 years and set revision rates to average revision rates instead of age-adjusted revision rates. Gademan reports a reduction of revision TKAs of 17%. Applying the same settings as Gademan to our own model, results in a reduction of revision TKAs of 16%.

Even though the context and methods are different Rovers^5^, George^6^ and Dakin^7^ also calculate the cost-effectiveness of delayed or avoided TKA. We attempted to imitate their analyses to externally validate our own model. To do so, we 1.) identified outcomes that are quantified by their analyses as well as ours, 2.) identified key differences that could be replicated by settings in our models, 3.) identified key differences that could not be replicated in our model and 4.) tried to replicate results.

Rovers^5^ calculated the net-monetary loss as a result of postponing TKA by one week by multiplying the difference between pre- and post-surgery utility with the cost-effectiveness threshold and adding the cost per week. Rovers reports that postponing TKA with one week would lead to a net-monetary loss of 95€. We could not replicate these results using the formula and inputs that are used by Rovers. When trying to replicate the results of Rovers using Rovers’ inputs, the result was 85€. Using the same formula with the LROI inputs applied in our analyses, our analyses resulted in a net-monetary loss of 88€. The remaining differences between results can be explained by slightly different utility inputs (0.23 in our article vs 0.22 in Rovers’ article).

George^6^ quantified the value of postponing TKA without improvement of QoL in India. There are several key differences between the models: The article does not differentiate between early and late revisions, periprosthetic joint infections, arthrodesis, amputation into account. On the other hand, George does take mortality as a result of TKA into account. Notably, inputs also differ significantly due to the different contexts of the economic models. To emulate George’s article we set the QoL during the postponing period to that of patients with severe OA. As in George’s article we set the starting age of patients to 50 with a 20-year delay of TKA for all patients. Instead of the age-adjusted revision rate, we used an average revision rate for both intervention and comparator. George’s results suggest a similar cost (₹242 618 for the intervention and ₹247 256 for the comparator) and a larger gap in the QALYs (14.62 QALYS for the intervention and 16.71 QALYs for the comparator). Our analyses with similar settings to George resulted in large gaps in costs (€8,674 for the intervention and €19,191 for the comparator) and in effects (14.24 QALYs for the intervention and 17.57 QALYs for the comparator). While the effects of postponing TKA on costs differ substantially between the analyses, the consequence of postponing TKA on health seems to be relatively similar. The difference in costs between analyses can be explained by the healthcare resource use. Our analyses did not apply any healthcare resource use for the intervention period, while George’s analyses applied a healthcare resource use for the intervention period which is roughly double of the cost after a TKA.

Dakin^7^ conducted a trial-based economic analysis to restrict TKA to patient subgroups with more severe OA. The main analyses of the model (restricting TKA by severity) could not be reproduced with our article. The results of analyses restricting TKA for the average patients reported in the discussion and key message section of the article could be emulated in our model. As in the George article, the inputs and intermediary results differ substantially, with OS utility being as low as 0.39 (0.55 in our article), utility after 1 year of TKA being 0.71 (0.8 in our article), costs for primary TKA being £6,363 (€9,650 in our article) and health state costs over 5 years post TKA costing £1,095 (€3,474 in our article). To emulate the analyses of Dakin, we applied a 5-year time horizon, a 5-year delay of TKA for all patients, no improvement in utility while TKA was postponed and a healthcare perspective. With these settings our analyses resulted in an ICER of €17,477/QALY which is substantially higher than the £5623/QALY reported in Dakin’s article. This is unsurprising as the utility benefit is larger and costs for TKA are lower, possibly simply owing to a different setting.

There are differences between the results of our article and the results the three articles which we found to be comparable. These differences can to a large extent be explained by differences in inputs and methods, owing to different aims and contexts of the analyses. Despite the differences, the mechanisms and conclusions in all analyses are similar: Delaying TKA even with strong conservative assumptions (no costs in the OA health state, no further utility deterioration of OA when left untreated, short duration of delay) is not cost-effective without improvement of the patient’s quality of life.

**References**

1. Husereau D, Drummond M, Augustovski F, de Bekker-Grob E, Briggs AH, Carswell C, et al. Consolidated Health Economic Evaluation Reporting Standards (CHEERS) 2022 Explanation and Elaboration: A Report of the ISPOR CHEERS II Good Practices Task Force. Value Heal [Internet]. 2022;25(1):10–31. Available from: https://doi.org/10.1016/j.jval.2021.10.008

2. Büyükkaramikli NC, Rutten-van Mölken MPMH, Severens JL, Al M. TECH-VER: A Verification Checklist to Reduce Errors in Models and Improve Their Credibility. Pharmacoeconomics [Internet]. 2019;37(11):1391–408. Available from: https://doi.org/10.1007/s40273-019-00844-y

3. Dutch Arthroplasty Register. LROI Annual report 2022. 2022;(December 2021):1–214.

4. Gademan MGJ, Van Steenbergen LN, Cannegieter SC, Nelissen RGHH, Marang-Van De Mheen PJ. Population-based 10-year cumulative revision risks after hip and knee arthroplasty for osteoarthritis to inform patients in clinical practice: a competing risk analysis from the Dutch Arthroplasty Register. Acta Orthop. 2021;92(3):280–4.

5. Rovers MM, Wijn SRW, Grutters JPC, Metsemakers SJJPM, Vermeulen RJ, Van Der Pennen R, et al. Development of a decision analytical framework to prioritise operating room capacity: lessons learnt from an empirical example on delayed elective surgeries during the COVID-19 pandemic in a hospital in the Netherlands. BMJ Open. 2022;12(4):1–10.

6. George J, Gautam D, Devasenapathy N, Malhotra R. Is It Worth Delaying Total Knee Replacement as Late as Possible? A Cost-Effectiveness Analysis Using a Markov Model in the Indian Setting. Value Heal Reg Issues [Internet]. 2021;24:173–80. Available from: https://doi.org/10.1016/j.vhri.2020.12.009

7. Dakin H, Gray A, Fitzpatrick R, MacLennan G, Murray D. Rationing of total knee replacement: A cost-effectiveness analysis on a large trial data set. BMJ Open. 2012;2(1):1–9.
